# Investigation of PbSnTeSe High-Entropy Thermoelectric Alloy: A DFT Approach

**DOI:** 10.3390/ma16010235

**Published:** 2022-12-27

**Authors:** Ming Xia, Marie-Christine Record, Pascal Boulet

**Affiliations:** 1 Department of Chemistry, Aix-Marseille University, CNRS, IM2NP, 13007 Marseille, France; 2 Department of Chemistry, Aix-Marseille University, CNRS, MADIREL, 13007 Marseille, France

**Keywords:** thermoelectrics, high-entropy alloys (HEA), DFT calculations, transport properties

## Abstract

Thermoelectric materials have attracted extensive attention because they can directly convert waste heat into electric energy. As a brand-new method of alloying, high-entropy alloys (HEAs) have attracted much attention in the fields of materials science and engineering. Recent researches have found that HEAs could be potentially good thermoelectric (TE) materials. In this study, special quasi-random structures (SQS) of PbSnTeSe high-entropy alloys consisting of 64 atoms have been generated. The thermoelectric transport properties of the highest-entropy PbSnTeSe-optimized structure were investigated by combining calculations from first-principles density-functional theory and on-the-fly machine learning with the semiclassical Boltzmann transport theory and Green–Kubo theory. The results demonstrate that PbSnTeSe HEA has a very low lattice thermal conductivity. The electrical conductivity, thermal electronic conductivity and Seebeck coefficient have been evaluated for both n-type and p-type doping. N-type PbSnTeSe exhibits better power factor (PF = S^2^σ) than p-type PbSnTeSe because of larger electrical conductivity for n-type doping. Despite high electrical thermal conductivities, the calculated ZT are satisfactory. The maximum ZT (about 1.1) is found at 500 K for n-type doping. These results confirm that PbSnTeSe HEA is a promising thermoelectric material.

## 1. Introduction

In recent years, the energy problem has become increasingly serious. Thermoelectric materials have attracted extensive attention as they can directly convert waste heat into electric energy [1,2,3,4]. The figure of merit ZT = S^2^σT/κ [5] is used to evaluate the thermoelectric conversion efficiency in which S, σ, T, and κ are the Seebeck coefficient, electrical conductivity, temperature, and thermal conductivity (electronic and lattice), respectively. However, due to the strong coupling between these parameters, it is not easy to improve the thermoelectric efficiency [6]. Therefore, in order to improve the thermoelectric efficiency, various routes have been explored, e.g., using band engineering to improve power factor (S^2^σ) [7,8], and reducing the dimensionality of the material to reduce the lattice thermal conductivity [9,10,11]. In addition to improving the thermoelectric efficiency of existing materials with these strategies, finding new thermoelectric materials is also an important approach [12,13,14].

As a recent method of alloying, high-entropy alloys has attracted much attention in the fields of materials science and engineering [15,16,17,18,19,20]. A bibliographic search from the Chemical abstract Service/SciFinder database indicates that more than 11,000 papers have been published on high-entropy alloys (HEA) since 2002. Most of these references deals with materials containing mainly transition metal elements (Ti, Cr, Fe, Cu, Mo, Co, Ni, Nb, Ta, Pt, …) occasionally combined with metals or non-metals of the principal elements (Al, Si, P, …). These HEA have been investigated for, e.g., their mechanical (see Refs. [21,22,23,24]), catalytic [25,26,27,28], photocatalytic [29,30,31,32], and refractory [33,34,35,36] properties. To date, over 350 reviews have been published on HEAs various properties [37,38,39,40,41,42]. High-entropy alloys are typically defined as a single-phase, solid solution with five or more principal elements, each in a 5 to 35% molar ratio, resulting in high configurational mixing entropy (∆S_mix_), defined as ∆S_mix_ = −R∑_i_c_i_lnc_i_, where c_i_ and R are the compositional ratio and the gas constant, respectively [15]. It is generally admitted that the high entropy of mixing favors the formation of solid solutions and reduces the number of phases [15]. Because of the lattice distortion effects [43], which reduce phonon velocity and enhance the scattering of phonons, high-entropy alloys generally have low lattice thermal conductivity [44,45,46]. As high-entropy sulfides, Cu_5_Sn_1.2_MgGeZnS_9_ has been reported with a ZT value of 0.58 at 773 K [47]. The high-entropy metal chalcogenide (Ag,Pb,Bi)(S,Se,Te) alloy with a NaCl-type structure has been investigated and it was found that this compound is a n-type semiconductor with very low κ_L_ and good power factor resulting in a figure of merit of 0.54 at 723 K [48]. Recently, an n-type PbSe-based high-entropy material formed by entropy-driven structural stabilization was studied for its thermoelectric properties. The ZT value was found to reach 1.8 at 900 K, which corresponds to a material exhibiting good thermoelectric properties [49]. Apart from HEA, other, more conventional, types of compounds have been reported bearing low thermal conductivity, such as Zintl phases (e.g., Ba_2_ZnSb_2_ [50]), argyrodites [51], sulfide-containing films [52], rare-earth molybdates [53], perovskites (e.g., [54]), and defective metal chalcogenide thin films [55], to cite a few. Typically, the thermal conductivity of these compounds lies below 1 W/(m K). The reasons, or the conjunction of reasons, for this low thermal conductivity have been identified by means of the combined density-functional theory and Boltzmann transport theory approaches. The investigations have evidenced large Grüneisen parameters, low-lying optical and acoustic phonon frequencies, short phonon lifetime, and the presence of defects. We shall mention that, to date, several papers report on the prediction of the thermal conductivity of materials using a combined approached based on ab initio molecular dynamics used to train a machine-learned force field subsequently used with the Green–Kubo formalism to derive the heat flux and thermal conductivity [56,57,58]. These approaches are similar to the one used in this work, which is based on the work from Verdi et al. [59]. It has been shown that this machine-learned approach is very effective and yields property data close to those obtained from the Boltzmann transport theory (see, e.g., Ref. [60]).

The fact remains that, so far, most of the research driven on the thermoelectric properties of high-entropy alloys are mainly experimental studies, theoretical studies on TE performance of high-entropy alloys being more seldom. Recently, PbSnTeSe has been experimentally investigated by Fan et al. [61] and Raphel et al. [62,63], who report appreciably similar results for the thermoelectric performance of the material (see below). The modelling of HEAs transport properties constitute a challenge, and both the simulation methods and their derived predictions have to be confronted to experimental data to assess their validity. In this context, we present in this paper for the first time a complete approach that allows one to obtain all the transport coefficients, including the figure of merit, of a high-entropy alloy. The thermoelectric transport properties of the PbSnTeSe high-entropy alloy have been calculated by combining state-of-the-art methods based on the special quasi-random structures approach to generate the HEA structure, the first-principles density-functional theory (DFT), and the Boltzmann transport and Green–Kubo theories. First, from the DFT-computed material electronic states, the electronic transport properties (electronic conductivity, Seebeck coefficient and electronic thermal conductivity) were calculated using the linearized Boltzmann transport equation, and the power factor was obtained. Next, the lattice thermal conductivity was evaluated according to the Green–Kubo (GK) theory [64] using on-the-fly machine-learned force fields (MLFF) [65,66]. Finally, the figure of merit of PbSnTeSe was determined. The results demonstrate that PbSnTeSe has very low lattice thermal conductivities. Despite high electrical thermal conductivities, the calculated ZT are satisfactory. The maximum ZT (about 1.1) is found at 500 K for n-type doping. These results confirm the interest of this HEA for thermoelectric applications.

## 2. Computational Details

Our computations were performed within the DFT framework utilizing the projector augmented wave (PAW) [67] technique, as implemented in the Vienna Ab Initio Simulation Package (VASP) [68,69,70]. The Perdew–Burke–Ernzerhof (PBE) functional under the generalized gradient approximation (GGA) [71] was utilized as the exchange-correlation functional. The nonempirical PBE functional is known to yield accurate crystal parameters and properties, and is known to fulfil many sum rules on the exchange-correlation hole [72]. Generally, where GGA functionals fail, local density functionals fail too. Concerning the thermal conductivity, compared to local density functionals, PBE does not over bind structures (it slightly under binds), hence the interatomic force constants are not too soft. Overall, the lattice thermal conductivity is quite the same whether using local density or GGA functionals [73].

The special quasi-random structures (SQS) of high-entropy alloys have been generated using the Monte Carlo SQS (MCSQS) tool as implemented in the alloy theoretic automated toolkit (ATAT) [74]. A 2 × 2 × 2 supercell consisting of 64 atoms was built for calculations. A 3 × 3 × 3 Monkhorst–Pack k-point mesh was used, and the kinetic energy cutoff was set to 400 eV. The geometric structures were totally relaxed until the Hellmann-Feynman forces were less than 0.01 eV/Å. The electronic transport properties were computed with the Boltztrap2 code, which implements the semiclassical Boltzmann theory under the relaxation time approximation (RTA) [75]. As the calculation of transport coefficients is very demanding in term of the band structure accuracy, a much denser, 9 × 9 × 9 k-point mesh was employed to obtain the electrons energy eigenvalues for the subsequent electronic transport properties calculation. As within the RTA the lifetime of electrons has to be determined separately, which constitutes an inherent limitation to the linearized Boltzmann transport equation approach, the relaxation time (τ) was determined using the deformation potential (DP) theory [76]. Due to heavy elements present in the structure, the spin–orbit coupling (SOC) effect was considered in our calculations. The lattice thermal conductivity was calculated using the Green–Kubo theory for which the heat flux was obtained from the on-the-fly machine-learned force fields [65,66] module of VASP. The evident advantage of using this approach is that classical molecular dynamics allows for catching the phonon dynamics at any order and is applicable to potentially extremely large structures. The difficulty is that interatomic parameters of good quality have to be available. The first step of the MLFF elaboration consists in training the force field by machine learning through molecular dynamics (MD) simulations in the NVT ensemble, where N, V, and T are the number of particles, volume and temperature, respectively, that are kept constant during the simulations (canonical ensemble). A supercell of 512 atoms was used for the training, and the MD simulations were run with a time step of 1 fs. Then, after training, the force field was used to equilibrate the system in the NVT ensemble at the desired temperature with a time step of 1 fs for 100 ps. Finally, the heat flux was calculated through molecular dynamics simulations in the NVE ensemble, where N, V and the energy E are kept constant (microcanonical ensemble), with a time step of 1 fs for 100 ps. For the ensemble average, 10 independent molecular dynamics simulations were performed for the calculation of the heat flux.

The design of the force-field (FF) is described in detail in Ref. [59]. We give a brief summary here. The fitting of the force-field parameters relies on the availability of a database (DB) of structures, the quality of the fitting being assessed from energies, atomic forces, and stress tensors. This DB is built on-the-fly through the ab initio molecular dynamics (AIMD) simulations. At each step of the AIMD a decision is made as to whether a new AIMD step should be run to add a new structure to the DB or if the MD step is run with the FF. The decision is made after the estimated errors between the ab initio atomic forces and the FF ones based on a Bayesian inference. Hence, the algorithm relies on the Bayesian linear regression to assess the quality of the FF parameters. In our case, the force-field parameters were built from a DB containing more than 2100 structures. The Bayesian error on the atomic forces, energies and stress are below 0.006 eV/Å, 0.5 meV and 0.04 kB, respectively. The FF parameters quality is assessed based on the capability of the FF to reproduce properly the two-body and three-body distributions (Equations (2) and (3) in Ref. [59]) that represent the likelihood to find, around a given atom, an atom at a certain distance or a pair of atoms at a certain distance and angle, respectively. Thereupon, the calculated cell parameters with the FF yields are the same as those obtained ab initio.

## 3. Results and Discussion

### 3.1. Structural and Electronic Properties of PbSnTeSe High-Entropy Alloy

**Figure 1:**
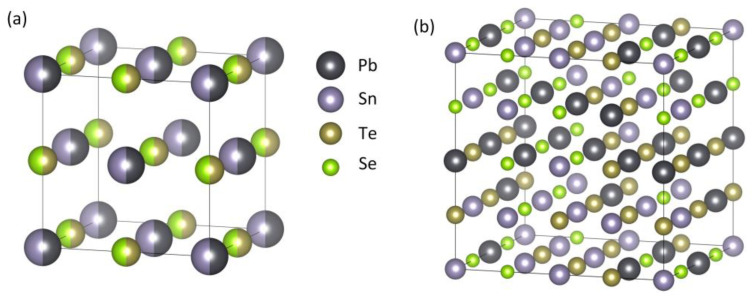
(**a**) Schematic view of the crystal structure of PbSnTeSe. (**b**) SQS generated by ATAT.

The PbSnTeSe high-entropy alloy is based on the NaCl-type face-centered cubic (FCC) crystal structure and a 2 × 2 × 2 supercell containing 64 atoms was constructed. Figure 1a shows the schematic illustration of the crystal structure of the PbSnTeSe HEA. The HEA structure was built from the PbTe crystal structure for which the same atomic ratio of 50% was employed for both Pb and Sn on the Pb Wyckoff positions, and Te and Se on the Te Wyckoff positions. Figure 1b shows the special quasi-random structure of PbSnTeSe high-entropy alloys generated by the MCSQS tool of ATAT. The optimized lattice parameters of the PbSnTeSe are a = 12.74 Å, b = 12.65 Å, and c = 12.68 Å, α = 89.8°, β = 90.2°, and γ = 90.6°.

**Figure 2:**
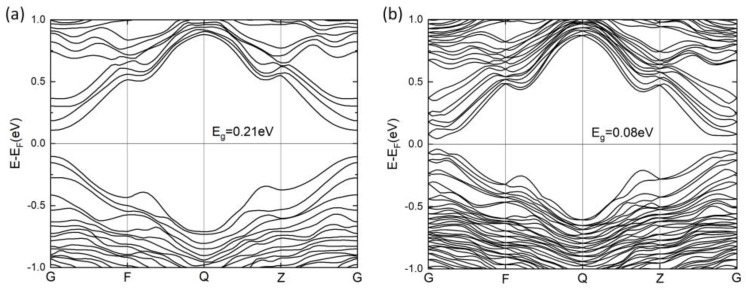
Calculated band structures of PbSnTeSe without spin–orbit coupling (SOC) (**a**) and with SOC (**b**).

The band structure of PbSnTeSe was calculated along the line (0 0 0)-(0 ½ 0)-(0 ½ ½)-(0 0 ½)-(0 0 0) of the Brillouin zone and is depicted in Figure 2. PbSnTeSe is a semiconductor with a direct band gap. Without spin–orbit coupling, the valence-band maximum (VBM) and the conduction-band minimum (CBM) are both located at the Γ point. Because of the heavy elements Pb, Te and Sn, the spin–orbit coupling effect was accounted for. The band gap decreases from 0.21 eV to 0.08 eV when considering the SOC effects, and the direct bandgap shifts slightly away from Γ. In addition, the SOC lifts the degeneracy of the crystal states leading to two states (j = l ± ½) between the high symmetry k-points, which can be explained by the main contribution of the *p* atomic orbitals of the atoms.

### 3.2. Seebeck Coefficient, Electrical Conductivity, and Power Factor of PbSnTeSe

**Figure 3:**
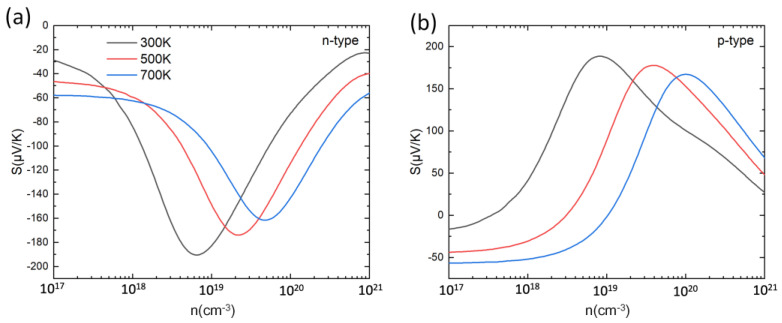
Seebeck coefficient (S) for n-type (**a**) and p-type (**b**) PbSnTeSe at 300 K, 500 K and 700 K as a function of carrier concentration.

Based on the calculated electronic structure, the Seebeck coefficient (S) of PbSnTeSe was determined. Figure 3 shows the Seebeck coefficient at 300 K, 500 K, and 700 K as a function of carrier concentration. For both n-type and p-type doping, the Seebeck coefficients first rise then decrease as the carrier concentration increases, which can be interpreted from the Mott formula [77]

(1) $$S=\frac{8\pi^{2}k_{B}^{2}}{3eh^{2}}m_{d}^{*}T{\left(\frac{\pi }{3n}\right)}^{\frac{2}{3}}$$

where *h*, *k_B_*, m*_d_, *T* and *n* are the Planck constant, Boltzmann constant, density of states effective mass and carrier concentration, respectively. According to this expression, apart from the effect of temperature, the Seebeck coefficient is governed by the ratio m*_d_ n^−2/3^. Assuming the simple evolution of the density of states (DOS) for 3D materials as the square-root of the state energies (see, e.g., Figure 39.1 in Ref. [78]), at low doping level the curvature radius increases drastically and hence the DOS mass, and overall, the Seebeck coefficient increases sharply. As the doping level increases further, the DOS mass becomes roughly constant, and the *n*^−2/3^ term starts dominating in the Mott formula, leading to a decrease of S. For both low and highly doped compound (*n* ~ 10^17^ cm^−3^ and *n* ≥ 10^21^ cm^−3^), the Seebeck coefficient is improved with the increase in the temperature, which can also be understood by this formula. In the meantime, the maximum S values are reduced with the temperature increase. The peak values of S for PbSnTeSe are all in the range of 160–190 μV/K with little difference between n-type and p-type.

As in RTA the electrical conductivity is scaled by the carrier relaxation time *τ*, this parameter has to be determined to get the values of σ. For this, the deformation potential (DP) theory [76] was utilized, from which the expression of *τ* reads:

(2) $$\tau =\frac{2\sqrt{2\pi }\hbar^{4}C}{3{\left(k_{B}Tm^{*}\right)}^{\frac{3}{2}}E_{1}^{2}}$$

where ℏ and T are the reduced Planck constant and temperature, respectively. The effective mass of the carrier is calculated by $m^{*}=\hbar^{2}/\left(\partial^{2}E/\partial k^{2}\right)$, and the elastic constant is defined as $C=\left[\partial^{2}E/\partial {\left(\Delta a/a_{0}\right)}^{2}\right]/V_{0}$ where E, Δa and $V_{0}$ are the total energy of the system, the change of the lattice parameter and the equilibrium volume, respectively. The DP constant $E_{1}$ corresponds to the shift of the band edge energy and is given by $E_{1}=\partial E_{edge}/\partial \left(\Delta a/a_{0}\right)$, where $E_{edge}$ is the band edge energy. The calculated τ values are listed in Table 1. At the temperatures of 300 K, 500 K, and 700 K, the relaxation times for n-type and p-type PbSnTeSe vary from 158 to 44.3 fs and from 17.3 to 4.78 fs, respectively. The relaxation time for the n-type PbSnTeSe is larger than that of the p-type because the effective mass and deformation potential of the n-type compound are smaller than those of the p-type. The decrease in the relaxation time with increasing temperature means that the scattering of carriers is gradually enhanced. The scattering inhibits the transport of carriers, which should lead to a decrease of the conductivity with increasing temperature.

**Figure 4:**
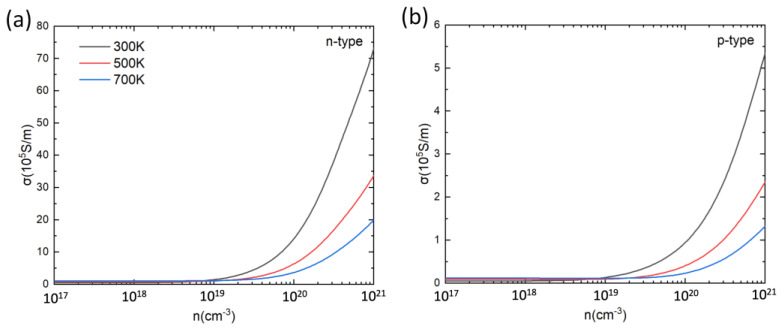
Electrical conductivity (σ) for n-type (**a**) and p-type (**b**) PbSnTeSe at 300 K, 500 K and 700 K as a function of carrier concentration.

Based on the calculated relaxation time, the electrical conductivity (*σ*) of PbSnTeSe was obtained. Figure 4 shows the calculated *σ* at 300 K, 500 K and 700 K as a function of the carrier concentration. For both n-type and p-type doping, σ increases with the increase in carrier concentration. For low carrier concentrations σ increases with temperature, and for high concentrations (>10^19^ cm^−3^) σ decreases with the increase in temperature, which can be understood from the Drude–Sommerfeld formula [79,80,81]:

(3) σ=neμ

(4) $$\mu =\frac{\tau e}{m^{*}}$$

where n is the carrier concentration and *μ* is the mobility of the carriers. As mentioned above, with the temperature increase the relaxation time decreases, which leads to a decrease in mobility, hence the conductivity decreases as the temperature increases. In each case, the n-type PbSnTeSe has a better electrical conductivity than the p-type, which is caused by a larger relaxation time for the n-type than for the p-type PbSnTeSe.

**Figure 5:**
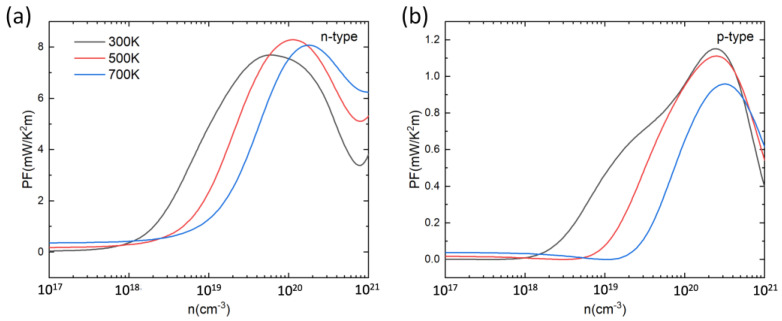
Power factor (PF) for n-type (**a**) and p-type (**b**) PbSnTeSe at 300 K, 500 K and 700 K as a function of carrier concentration.

Based on the calculated S and σ, the power factor PF ($S^{2}\sigma$) was determined. Figure 5 shows the calculated PF at 300 K, 500 K, and 700 K as a function of carrier concentration. For both n-type and p-type doping, the PF first increases then decreases with the increase in carrier concentration. The peak values of PF for p-type and n-type PbSnTeSe are in the range of 0.9–1.2 mW/(m K^2^) and 7–8 mW/(m K^2^), respectively. In each case, the optimal PF values for n-type doping are larger than those for p-type doping because of the higher electrical conductivity, indicating that n-type doping is more efficient than the p-type at improving the TE performance of PbSnTeSe.

### 3.3. Electronic Thermal Conductivity of PbSnTeSe

**Figure 6:**
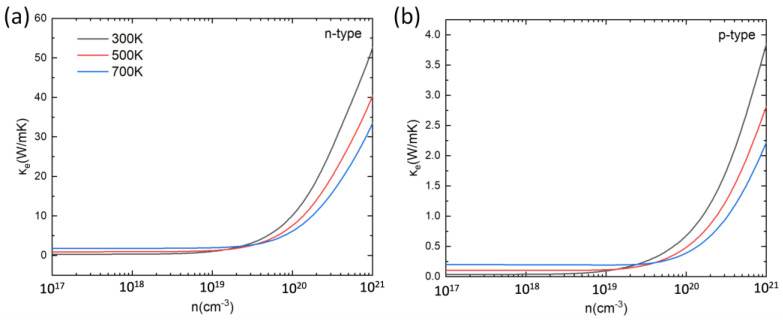
Electronic part of the thermal conductivity (κ_e_) for n-type (**a**) and p-type (**b**) PbSnTeSe at 300 K, 500 K, and 700 K as a function of carrier concentration.

Thermal conductivity is composed of two parts, the electronic thermal conductivity ($k_{e}$) and the lattice thermal conductivity ($k_{L}$). The electronic thermal conductivity was calculated using the Wiedemann–Franz law [82,83],

(5) $$k_{e}=L\sigma T$$

where *L* was approximated by the Lorenz number *L*_0_ that takes the value 2.44 × 10^8^ WΩK^−1^. Whereas the deviation of the *L*/*L*_0_ ratio from one is still an open question for nanoscale materials [84], it seems that the deviation from the Wiedemann–Franz law occurs mainly at low temperature (well below 300 K) where lattice vibrations increase the *L/L*_0_ ratio above 1. However, this tendency can also be counteracted by electronic corrections, leading finally to a small change of the *L/L*_0_ ratio (between 0.8 and 1.2, at most). For bulk compounds, the same effects can occur. Therefore, we are confident that the conclusions presented hereafter should not change drastically with *L*. Figure 6 shows the calculated $k_{e}$ at 300 K, 500 K, and 700 K as a function of the carrier concentration. Due to the linear correlation between $k_{e}$ and σ, the impact of carrier concentration and temperature on the electronic thermal conductivity is the same to that on the electrical conductivity. For both n-type and p-type doping, $k_{e}$ increases with the increase in carrier concentration, and increases (decreases, resp.) with temperature for low (high, resp.) carrier concentrations. In each case, the n-type PbSnTeSe has a larger $k_{e}$ than the p-type.

### 3.4. Machine-Learned Force-Field Potential

**Figure 7:**
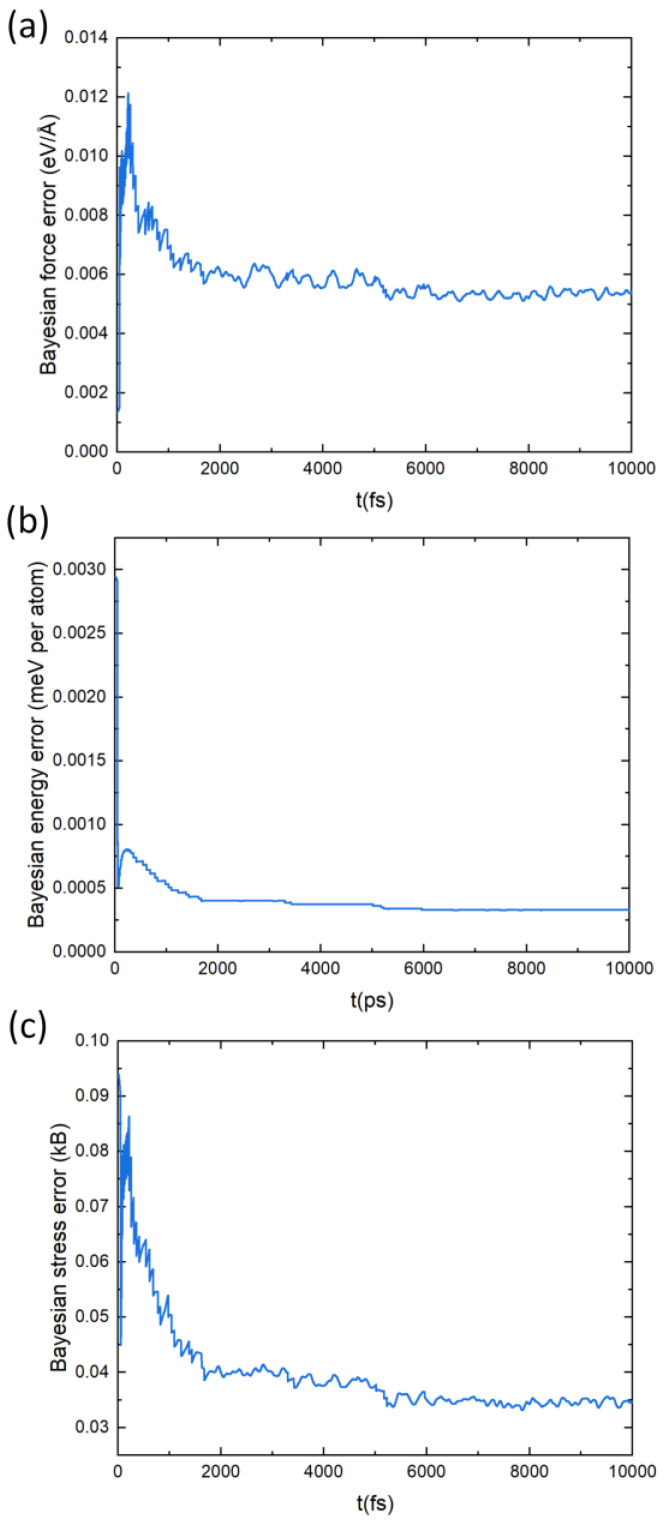
Estimated Bayesian error in the forces (**a**), energies (**b**), and stress tensors (**c**) predicted by the MLFF for the training dataset.

To build an interatomic potential force field (FF) for PbSnTeSe the on-the-fly machine-learned FF algorithm integrated in the VASP code was used. The training strategy of MLFF consists in constructing the force field on the fly during MD simulation and the predicted Bayesian error is used at every MD step to judge whether additional first-principle calculations need to be performed and a new structure be included in the dataset or not. When the force field is trained by the on-the-fly machine-learning algorithm, many of the MD steps are carried out with the force field, and first-principles calculations are executed only when the predicted Bayesian error is large. Figure 7 depicts the estimated Bayesian error of the MLFF, which shows that it is consistently lowering. Table 2 shows the root-means-square errors (RMSE) in the energies, forces, and stress tensors predicted by MLFFs for the training dataset. The predicted errors are low, which agrees with the general observation that the Bayesian linear regression (BLR), which was adopted to obtain the regression coefficients of kernel-based methods, leads to lower errors than other methods [59]. The lattice parameters calculated with the MLFF are a = 12.72 Å, b = 12.64 Å, c = 12.67 Å, α = 89.9°, β = 90.1° and γ = 90.2°, which are very close to those calculated with PBE.

### 3.5. Lattice Thermal Conductivity of PbSnTeSe

**Figure 8:**
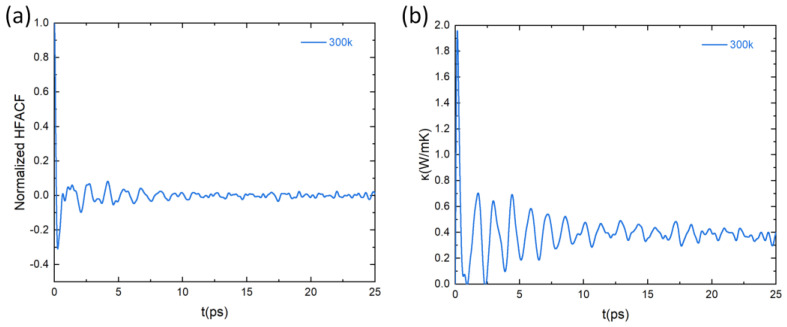
Lattice thermal properties at 300 K from GK theory. (**a**) Heat-flux autocorrelation function (HFACF) normalized by its zero-time value and (**b**) lattice thermal conductivity as a function of correlation time at T = 300 K.

According to the Green–Kubo theory, the thermal conductivity and the heat flux are related by [64]:

(6) $$\kappa =\underset{t\to \infty }{\lim}\frac{1}{3k_{B}T^{2}V}\int_{0}^{t}<j\left(t^{\prime }\right)j\left(0\right)>dt^{\prime }$$

where $k_{B}$, T and V denote the Boltzmann constant, the temperature, and the volume of the system, respectively. $j\left(t\right)$ is the heat flux and the symbols $<\cdot >$ represent the ensemble average over every MD simulation. Based on the heat flux calculated by the MLFF, the heat-flux autocorrelation function (HFACF) of PbSnTeSe was obtained. Figure 8a shows the calculated normalized averaged HFACF at the temperature of 300 K as a function of correlation time. At the beginning, the HFACF starts at one and rapidly drops to oscillate around zero. As correlation time increases, the oscillation gradually decreases, and finally approaches zero, indicating that the HFACF has converged. The HFACF is then used to determine the thermal conductivity ($\kappa_{L}$) of PbSnTeSe. Figure 8b shows the calculated $\kappa_{L}$ at the temperature of 300 K as a function of correlation time. The trend of $\kappa_{L}$ over correlation time is the same as that of HFACF. Initially, the oscillation is relatively large and finally tends to stabilize converging towards a constant value, hence asserting the proper convergence of our simulations.

**Figure 9:**
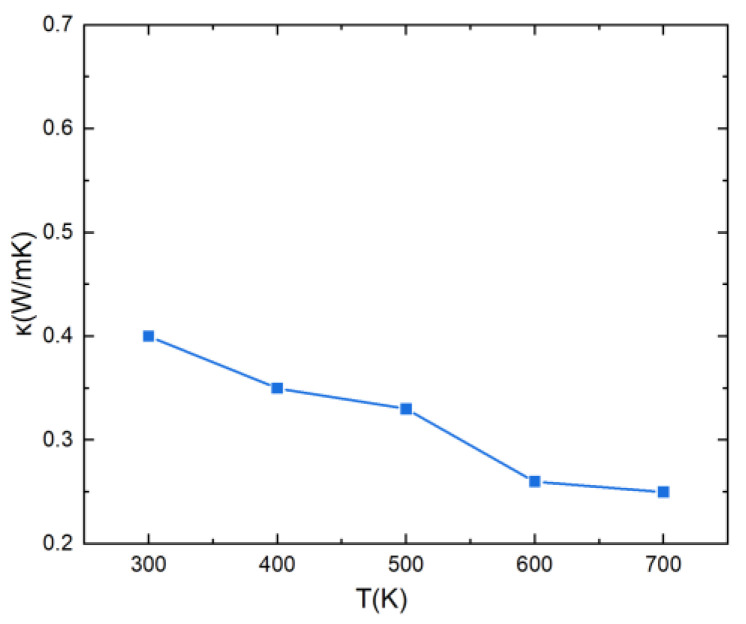
Lattice thermal conductivity of PbSnTeSe as a function of temperature.

Using the same approach, the value of $\kappa_{L}$ was computed for various temperatures in the range 300–700 K (Figure 9). At 300 K, the $\kappa_{L}$ value is 0.4 W K^−1^ m^−1^, which is a very low value of the lattice thermal conductivity, favorable for thermoelectric materials. This is due to the lattice distortion effect of high-entropy alloys that can reduce phonon velocity and enhance phonon scattering, resulting in low thermal conductivity. Additionally, one can observe that when the temperature rises, the thermal conductivity of the lattice decreases, which is also due to an increase in phonon scattering at higher temperatures.

### 3.6. Figure of Merit of PbSnTeSe

**Figure 10:**
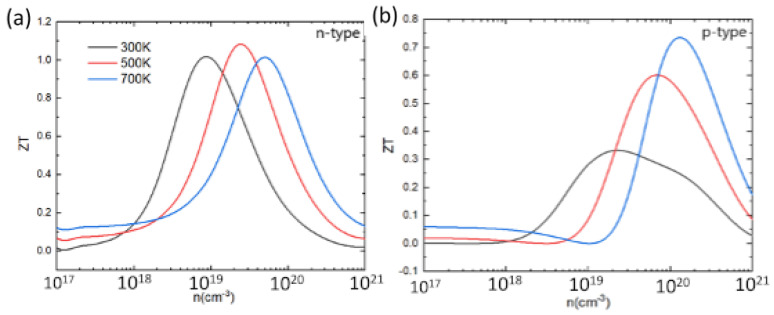
Figure of merit (ZT) for n-type (**a**) and p-type (**b**) PbSnTeSe at 300 K, 500 K, and 700 K as a function of carrier concentration.

Based on the electronic and thermal transport coefficients, the ZT value can be determined. Figure 10 shows the calculated ZT at 300 K, 500 K, and 700 K as a function of the carrier concentration. For both p-type and n-type PbSnTeSe, the ZT value first increases to an optimal value then decreases with increasing concentration. The peak value of ZT for n-type does not vary significantly as the temperature rises, only the ideal carrier concentration shifts a little towards higher values. However, for p-type, the peak of ZT increases significantly with increasing temperature, and the ideal carrier concentration shifts to higher values. The best ZT value for n-type PbSnTeSe is 1.1 at 500 K, while for p-type doping it amounts to 0.75 at 700 K. At each temperature, the ZT value for n-type compound is greater than that of p-type, mainly resulting from the large electrical conductivity for n-type doping, indicating that n-type PbSnTeSe exhibits better thermoelectric properties than the p-type.

### 3.7. Comparison with Available Data on PbSnTeSe and Other HEA

As mentioned in the introduction section, Fan et al. [61] and Raphel et al. [62,63] have reported experimental investigations on the thermoelectric properties of PbSnTeSe. The transport coefficients reported by these groups are presented in Table 3. We first note that the experimental figures of merit ZT are lower than ours by a factor of around two. To trace back the origin of this difference, we compare the predicted transport coefficients at 700 K and for the carrier concentration in electrons of 6 × 10^19^ e/cm^−3^ with the experimental results from Fan et al. The calculated total thermal conductivity (2.3 W/(mK)) is higher than that obtained experimentally, which should degrade the theoretical ZT value, but this is not what we observe. By contrast the calculated power factor S^2^σ is much higher (almost eight times as high). As our Seebeck coefficient is the same as the experimental one, the reason for the difference is to be found in the electrical conductivity. Indeed, σ_theo_ amounts to 15 × 10^4^ S/m. This large value can be explained by two factors. First, the calculated gap that includes the spin–orbit interaction is smaller by a factor of three than the experimental result, and second, we are modeling a pure, defect-free compound. In real compounds, electrons are scattered by impurities, defects, and grains boundaries, which are not accounted for in our model.

Recently, Bafekry et al. [85] have reported thermoelectric properties for the GeSnPbSSeTe HEA. Their investigation is limited to the electronic transport properties (Seebeck coefficient and electrical and electronic thermal conductivities) but the electrons relaxation time was not determined. Interestingly, the Seebeck coefficient for the n-doped compound is very similar to that of PbSnTeSe (around −150 μV/K). Assuming about the same value of the electron relaxation time the electrical conductivity of GeSnPbSSeTe is of the same order of magnitude (~20 × 10^4^ S/m) as that of PbSnTeSe. Assuming further that the thermal conductivity is similar for both compounds, one can infer that these compounds perform equally. From the experimental side [86], GeSnPbSSeTe shows a similar Seebeck coefficient, but the electrical conductivity is much lower, being of the order of 600 S/m.

As a different HEA, Sn_0.25_Pb_0.25_Mn_0.25_Ge_0.25_Te was investigated both experimentally and theoretically by Wang et al. [87]. A ZT value of 1.0 was found at 700 K, probably due to a drastic decrease in the thermal conductivity down to 0.76 W/(mK) by entropy engineering, compared to SnTe (4 W/(mK)). Compared to PbSnTeSe, the Seebeck coefficient of Sn_0.25_Pb_0.25_Mn_0.25_Ge_0.25_Te is twice as small (~100 μV/K), but the power factor is notably higher (14 × 10^−4^ W/(mK^2^)). It was indeed observed that the electrical conductivity increases with alloying with more elements. This result shows that, PbSnTeSe could be an interesting candidate for thermoelectric application as a high-entropy alloy materials, but there is probably room for improvement, in particular on the electrical conductivity by further alloying with other elements.

## 4. Conclusions

In summary, by combining first-principles calculations and on-the-fly machine learning technique with the semiclassical Boltzmann transport theory and Green–Kubo theory, the thermoelectric transport properties of PbSnTeSe high-entropy alloy have been thoroughly investigated. The electronic and thermal transport coefficients of PbSnTeSe high entropy-alloy have been discussed in detail. The results indicate that PbSnTeSe has very low lattice thermal conductivities, below 0.4 W K^−1^ m^−1^. It has been found that the PF values for n-type doping are always larger than those for p-type doping because of the higher electrical conductivity. The n-type PbSnTeSe exhibits better thermoelectric properties than the p-type. The maximum ZT (≈1.1) is found at 500 K for n-type doping. These results confirm that the PbSnTeSe HEA is a promising thermoelectric (TE) material.

## Figures and Tables

**Table 1:** Elastic constant *C*, deformation potential *E_1_*, effective mass m* and relaxation times *τ* at 300 K, 500 K and 700 K of PbSnTeSe.

Carrier Type	*C*	*E_1_*	m*	*τ* (fs)	*τ* (fs)	*τ* (fs)
	eV/Å^3^	(eV)	(m_e_)	300 K	500 K	700 K
Hole	0.195	12.945	0.520	17.3	8.06	4.87
Electron	0.195	6.434	0.303	158	73.4	44.3

**Table 2:** Root-means-square errors in the energies, forces, and stress tensors predicted by the MLFF for the training dataset.

Energy (meV/Atom)	Force (eV/Å)	Stress (kB)
1.045	0.057	0.244

**Table 3:** Experimental thermoelectric properties of PbSnTeSe (from Refs. [61,62]). The data from Fan et al. [61] are at 700 K and for n = 6 × 10^19^ e/cm^−3^, and those from Raphel et al. [62] are at 625 K.

Property	Fan et al. [61]	Raphel et al. [62]
Seebeck coefficient (μV/K)	160	160
Electrical conductivity (S/m)	2.86 × 10^4^	2.65 × 10^4^
Power factor (W/(mK^2^))	8 × 10^−4^	6.7 × 10^−4^
Lattice thermal conductivity (W/(mK))	0.87	0.45
Total thermal conductivity (W/(mK))	1.2	0.9
Figure of merit	0.45	0.47

## Data Availability

Data available upon request.
